# The colorful mantle of the giant clam *Tridacna squamosa* expresses a homolog of electrogenic sodium: Bicarbonate cotransporter 2 that mediates the supply of inorganic carbon to photosynthesizing symbionts

**DOI:** 10.1371/journal.pone.0258519

**Published:** 2021-10-15

**Authors:** Mel V. Boo, Shit F. Chew, Yuen K. Ip

**Affiliations:** 1 Department of Biological Sciences, National University of Singapore, Singapore, Republic of Singapore; 2 Natural Sciences and Science Education, National Institute of Education, Nanyang Technological University, Singapore, Republic of Singapore; University of Cambridge, UNITED KINGDOM

## Abstract

Giant clams live in symbiosis with phototrophic dinoflagellates, which reside extracellularly inside zooxanthellal tubules located mainly in the colourful and extensible outer mantle. As symbiotic dinoflagellates have no access to the ambient seawater, they need to obtain inorganic carbon (C_i_) from the host for photosynthesis during illumination. The outer mantle has a host-mediated and light-dependent carbon-concentrating mechanism to augment the supply of C_i_ to the symbionts during illumination. Iridocytes can increase the secretion of H^+^ through vacuolar H^+^-ATPase to dehydrate HCO_3_^−^ present in the hemolymph to CO_2_. CO_2_ can permeate the basolateral membrane of the epithelial cells of the zooxanthellal tubules, and rehydrated back to HCO_3_^−^ in the cytoplasm catalysed by carbonic anhydrase 2. This study aimed to elucidate the molecular mechanism involved in the transport of HCO_3_^−^ across the apical membrane of these epithelial cells into the luminal fluid surrounding the symbionts. We had obtained the complete cDNA coding sequence of a homolog of *electrogenic Na*^*+*^*-HCO*_*3*_^*−*^ *cotransporter 2* (*NBCe2-like gene*) from the outer mantle of the fluted giant clam, *Tridacna squamosa*. *NBCe2-like* gene comprised 3,399 bp, encoding a protein of 1,132 amino acids of 127.3 kDa. NBCe2-like protein had an apical localization in the epithelial cells of zooxanthellal tubules, denoting that it could transport HCO_3_^−^ between the epithelial cells and the luminal fluid. Furthermore, illumination augmented the transcript level and protein abundance of *NBCe2-like* gene/NBCe2-like protein in the outer mantle, indicating that it could mediate the increased transport of HCO_3_^−^ into the luminal fluid to support photosynthesis in the symbionts.

## Introduction

Tropical waters are known as ‘deserts’ of the sea as they are poor in nutrients due to a lack of upwelling. However, some marine invertebrates such as scleractinian corals and giant clams can flourish in oligotrophic tropical waters. Giant clams (Genus: *Tridacna* or *Hippopus*) are inhabitants of the Indo-Pacific reef ecosystems and are the largest of all bivalves. Despite the shortage of nutrients, giant clams grow rapidly because they can establish a mutualistic relationship with Symbiodiniaceae dinoflagellates of genera *Symbiodinium*, *Cladocopium*, *Durusdinium* and *Gerakladium* [1–6]. In giant clams, symbiotic dinoflagellates reside extracellularly inside a branched tubular system that originates from the host’s digestive tract [7]. The tertiary tubules that hold the majority of symbionts are located predominantly in the colorful and extensible outer mantle that contains pigments and iridophores [7]. Iridophores are aggregates of iridocytes that can deflect light of relevant wavelength to the symbiotic dinoflagellates to promote photosynthesis [8]. They can also absorb harmful UV radiation [9] during insolation. Photosynthesizing dinoflagellates can donate as much as 95% of photosynthates to the clam host to fulfil its energy and nutrition requirements [10], and to support high growth rate with light-enhanced shell formation [11,12]. In return, the host provides the symbionts with essential nutrients such as inorganic carbon (C_i_), phosphorus, and nitrogen to facilitate their growth and metabolism [13–19].

Symbiotic dinoflagellates need an increased supply of C_i_ to conduct C3 photosynthesis catalyzed by form II ribulose-1,5-bisphosphate carboxylase/oxygenase (RuBisCO) [20,21] during illumination. As they do not have access to the ambient seawater, photosynthesizing symbionts can deplete C_i_ in the hemolymph of giant clams in <13 min when there is no replenishment by respiratory CO_2_ or exogenous C_i_ [22]. Hence, the host clam must increase the absorption of C_i_ from the external medium and supply it to the photosynthesizing symbionts in the colorful outer mantle [19,21]. It has been established that the ctenidium (gill) of the fluted giant clam, *Tridacna squamosa*, expresses dual domain carbonic anhydrase (DDCA) [14], Na^+^/H^+^ exchanger 3 (NHE3) [23] and vacuolar H^+^-ATPase (VHA) [24] that can act together and constitute a light-dependent mechanism for Ci absorption. In fact the gene and protein expression levels of these three transporters are upregulated in the ctenidium during light exposure. Subsequently, the absorbed C_i_ is translocated as HCO_3_^−^ [22] through the hemolymph to other host organs, including the outer mantle that contain the majority of the extracellular symbionts.

C_i_ circulating in the hemolymph (as HCO_3_^−^) [22] must somehow permeate the basolateral (hemolymph-facing) membrane of the epithelial cells that form the zooxanthellal tubules. Then, C_i_ must get into the liminal fluid of the zooxanthellal tubules by crossing the apical (lumen-facing) membrane, so that it can be absorbed by the extracellular symbionts. The outer mantle of *T*. *squamosa* possesses a light-dependent carbon-concentrating mechanism (CCM) to augment the translocation of C_i_ from the hemolymph to the luminal fluid. This host-mediated CCM involves not only tubular epithelial cells but also iridocytes, with the participation of VHA [24] and carbonic anhydrase 2 (CA2) [25]. The iridocytes of the outer mantle of *T*. *squamosa* have a strong expression of VHA subunit A (ATP6V1A) [24]. As the transcript and protein expression levels of *ATP6V1A*/ATP6V1A increase in the outer mantle during illumination, there could be an increase in the iridocytes’ capacity to secrete H^+^ into the hemolymph during illumination. This could promote the dehydration of HCO_3_^−^ to CO_2_ in the hemolymph, and CO_2_ could permeate the basolateral membrane into the epithelial cells of the zooxanthellal tubules. Inside the epithelial cells, CO_2_ can be hydrated back to HCO_3_^−^, catalysed by the cytoplasmic CA2 [25], maintaining a favorable *P*CO_2_ gradient to augment the influx of CO_2_. Exposure to light also leads to a significant increase in the protein abundance of CA2 in the outer mantle of *T*. *squamosa* [25]. *A priori*, the apical membrane of these epithelial cells would express some sort of bicarbonate anion transporters (BATs) to transport HCO_3_^−^ from the cytoplasm to the luminal fluid of the zooxanthellal tubule, but such a transporter has not been identified [19].

It is noteworthy that the inhibition of anion transport with DIDS (4,4’-Diisothiocyanatostilbene-2,2’-disulfonate) impedes photosynthesis in scleractinian corals [26,27]. This suggests the involvement of BATs in the transport of HCO_3_^−^ to the intracellular endosymbionts in the oral gastroderm, but the exact mechanism of HCO_3_^−^ transport in scleractinian corals has not been elucidated. In mammals, two distinct families of BATs have been identified: solute carrier family 4 (SLC4) and solute carrier family 26 (SLC26). The SLC26 family comprises transporters that can transport diverse types of ion besides HCO_3_^−^, while the majority of BATs belongs to the SLC4 family [28]. Members of the SLC4 family can be categorized into three functional groups: (1) Na^+^-independent Cl^−^/HCO_3_^−^ exchangers (AE1-3); (2) Na^+^/HCO_3_^−^ cotransporters (NBCs), which can be either electrogenic (with the ‘e’ suffix; NBCe1 and NBCe2) or electroneutral (with the ‘n’ suffix; NBCn1 and NBCn2); and (3) Na^+^-driven Cl^−^/HCO_3_^−^ exchanger (NDCBE). In most cases, NBCe2 is described as an apical transporter in various epithelial cells [29–31] to facilitate HCO_3_^−^ efflux [31–33]. As no molecular information of NBCe2 is available in *T*. *squamosa*, this study was undertaken to clone and sequence a homolog of *NBCe2* (*NBCe2-like gene*) from the outer mantle of *T*. *squamosa*. Based on the deduced amino acid sequences, an anti-NBCe2-like polyclonal antibody was custom-made to examine the cellular and subcellular localization of NBCe2-like protein in the outer mantle of *T*. *squamosa* by immunofluorescence microscopy. If the putative NBCe2-like transporter were involved in delivering HCO_3_^−^ from the epithelial cells of the zooxanthellal tubule to the luminal fluid surrounding the symbionts, it should be localized at the apical membrane of these epithelial cells. In addition, quantitative real-time PCR (qPCR) and western blotting were performed to examine whether the expression levels of *NBCe2-like* gene and/or NBCe2-like protein were upregulated in the outer mantle of *T*. *squamosa* in response to light. It was hypothesized that these expression levels would increase during illumination in order to increase the capacity of HCO_3_^−^ translocation from the tubular epithelial cells to the luminal fluid in support of photosynthesis in the symbionts.

## Materials and methods

### Animals and experimental conditions

Adult *T*. *squamosa* weighing 520 ± 180 g (n = 23) were acquired from Xanh Tuoi Tropical Fish, Ltd (Ho Chi Minh City, Vietnam) and housed under a 12 h light:12 h dark regime for a duration of one month inside three glass tanks (length 90cm x width 62 cm x height 60 cm) at constant temperature (26 ± 1°C). The water conditions were as follows: temperature 26 ± 1°C; pH 8.1–8.3; salinity 30–32; hardness 143–179 ppm; calcium 280–400 ppm; phosphate < 0.25 ppm; nitrate 0 ppm; total ammonia < 0.25 ppm. The underwater light intensity (PPFD) at the level of the giant clams was 120 μmol photons m^-2^ s^-1^. This light intensity mimicked that received by *T*. *squamosa* in its natural habitat at a depth of ~ 20 m [34]. Institutional approval was not necessary for research on giant clams (National University of Singapore Institutional Animal Care and Use Committee). Five *T*. *squamosa* were subjected to darkness (n = 5; control), while 15 individuals were exposed to different light duration for 3, 6, or 12 h (n = 5 each light condition). Then, they were anaesthetized with 0.2% phenoxyethanol and sacrificed for tissue sampling. Samples of outer mantle were harvested and were freeze-clamped in liquid nitrogen, and were stored at -80°C prior to processing. Another three individuals, which had been exposed to light for 12 h (n = 3) were sacrificed for immunofluorescence microscopy.

### Extraction of mRNA and cDNA synthesis

TRI Reagent® (Sigma-Aldrich Co., St. Louis, MO, USA) was used to obtain the total RNA from the outer mantle of *T*. *squamosa*. Subsequently, the extracted total RNA was purified using PureLink RNA Mini Kit (Invitrogen). Procedures for the quantification of purified total RNA, RNA integrity, and cDNA synthesis were followed as described in [35].

### PCR, RACE-PCR, cloning and gene sequencing

The partial *NBCe2-like* cDNA sequence were isolated using a pair of PCR primers (forward: TTTACAGAGGAGAGCTTTGCC; reverse: CGGTAAGTCTCTGTTCCCT) designed from the homologous regions of *Oreochromis aureus NBCe2* (XM_031740849.1), *Chelonia mydas NBCe2* (XM_037886237.1) and *Homo sapiens NBCe2* (NM_001386136.1). PCR was performed using a 9902 Veriti 96-well thermal cycle (Thermo Fisher Scientific) with DreamTaq™ polymerase (Thermo Fisher Scientific Inc.). PCR was performed with an initial denaturation of 3 min at 95°C, followed by 40 cycles of denaturation, annealing and extension at 95°C for 30 s, 58°C for 30 s and 72°C for 1 min, respectively, and a final extension at 72°C for 10 min. PCR products were separated using agarose gel electrophoresis. Then, the band-of-interest was excised and purified using Wizard® SV Gel and PCR Clean-up System (Promega, Madison, WI, USA). The purified samples were prepared for sequencing using the BigDye® Terminator v3.1 Cycle Sequencing Kit (Thermo Fisher Scientific) and purified by ethanol/sodium acetate precipitation. Sequencing was performed using the 3130XL Genetic Analyzer (Thermo Fisher Scientific). Cloning was performed using pGEM®-T Easy Vector (Promega) following the methods of Hiong et al. [23]. Multiple clones were sequenced to obtain partial *NBCe2-like* sequences. The sequences were analyzed by BioEdit version 7.2.5. No isoforms were found. Subsequently, the full coding *NBCe2-like* sequence was obtained by performing RACE PCR with specific primers (Forward: 5’- CTTGGACATGCTAATCGTTGGTATTCTGG -3’ and Reverse: 5’- GTGAACTTTAGCTTCTTTCTGGATCCCAA -3’) using 5’ and 3’ RACE (SMARTer™ RACE cDNA amplification kit; Clontech Laboratories, Mountain View, CA, USA).

### Deduction of amino acid sequence

The *NBCe2-like* nucleotide sequence was translated into the NBCe2-like amino acid sequence using the ExPASy Proteomic server (http://web.expasy.org/translate/). TOPCONS protein structure prediction server was used to predict the transmembrane regions (TMs) of the deduced amino acid sequence (Tsirigos et al. 2015; http://topcons.cbr.su.se/). Glycosylation predictor (https://comp.chem.nottingham.ac.uk/glyco/) was used to predict N-glycosylation sites. The NBCe2-like amino acid sequence was deposited into GenBank with the accession number MW821489.

### Qualitative real-time PCR (qPCR)

cDNA (2 μg) was synthesized from the purified total RNA of the outer mantle of *T*. *squamosa* using random hexamer primers and RevertAidTM first strand cDNA synthesis kit. The absolute quantification of *NBCe2-like* transcripts was determined using a StepOnePlusTM Real-Time PCR System (Thermo Fisher Scientific) with a pair of specific qPCR primers (forward: 5’-GCTGTACCGACAAATATACGC-3’; reverse: 5’- GCAAAGTCGCTAACATTGGT-3’). The amplification efficiency for this primer set was 97.3%. The *NBCe2-like* transcript level was calculated using the plasmid standard curve of serially diluted plasmids, following the method of Hiong et al. [23,36].

### Antibodies

A rabbit polyclonal antibody against the NBCe2-like protein was custom-made by GenScript (Piscataway, NJ, U.S.A.) against residues 957–970 (DKDEKPSPPEKQTQ). This antibody was used for immunofluorescence microscopy and western blotting. The anti-α-tubulin antibody (12G10) used for western blotting was procured from the Developmental Studies Hybridoma Bank.

### Western blotting

Protein extraction for outer mantle samples was performed in accordance with the method of [23]. Proteins (100 μg) were separated by 6% SDS-PAGE and then trans-blotted onto a nitrocellulose membrane. Subsequently, the blots were incubated with the antibody raised against the anti-NBCe2-like protein (2 μg ml^−1^) or the anti-α-tubulin antibody (12G10, 0.05 μg ml^−1^) in Fast Western Antibody Diluent (Thermo Fisher Scientific Inc.) for 1 h at 25°C, following with secondary antibodies provided in the kit for 15 min at 25°C. The subsequent steps are as described in [35]. Peptide competition assay (PCA) was performed by incubating the anti-NBCe2-like antibody with the immunizing peptide (Genscript) at a ratio of 1:5 for 1 h prior to immunoblotting to confirm the specific band reactivity of the antibody.

### Immunofluorescence microscopy

The outer mantle samples were processed according to the method of [23]. Subsequently, the sections were stained with anti-NBCe2-like antibody (2 μg ml^-1^) overnight at 4°C. Then, the sections were incubated with Alexa Fluor 488, goat anti-rabbit (Invitrogen, 2.5 μg ml^-1^) secondary labeling in green at 37°C for 1 h. The sections were also stained with 4’6’-diamino-2-phenylindole (DAPI, Sigma-Aldrich Co.) to identify nuclei. Then, sections were mounted in ProLong Gold Antifade Mountant (Life Technologies, USA). Images were captured using a fluorescence microscope (Olympus BX60) equipped with a DP73 CCD digital camera (Olympus, Japan). Green fluorescence Alexa Fluor-488 was examined using the Olympus U-WNIBA Blue Fluorescence Filter (excitation wavelengths: 470–490 nm; emission wavelengths: 515–550 nm). Symbiotic dinoflagellates were examined for red autofluorescence of their plastids using the Olympus U-MWIG Interference Green Fluorescence Filter (excitation wavelengths: 520–550 nm; emission wavelengths: 580–800 nm). Images were captured under optimal exposure settings of 50–100 ms. Differential interference contrast (DIC) images of the tissue structures of both shell-facing and seawater-facing epithelium were produced using Olympus U-DICT DIC slider. All images were overlaid using Adobe Photoshop CC (Adobe Systems, San Jose, CA). PCA was performed by incubating the anti-NBCe2-like antibody with the immunizing peptide provided by Genscript at a ratio of 1:5 for 1 h prior to immunostaining.

### Statistical analysis

Statistical analysis was performed using IBM SPSS Statistics version 21 software (IBM Corporation, Armonk, NY, USA). Results were represent as means + SEM. The homogeneity of variance for the data sets was analyzed using Levene’s test. The differences between means of different data sets were evaluated using One-way analysis of variance (ANOVA), followed by either Tukey or Dunnett T3 post-hoc test, depending on the homogeneity of variance within the data sets. Differences were deemed statistically significant when the p-value was < 0.05.

## Results

### *NBCe2-like* nucleotide sequence and the deduced NBCe2-like amino acid sequence

**Fig 1:**
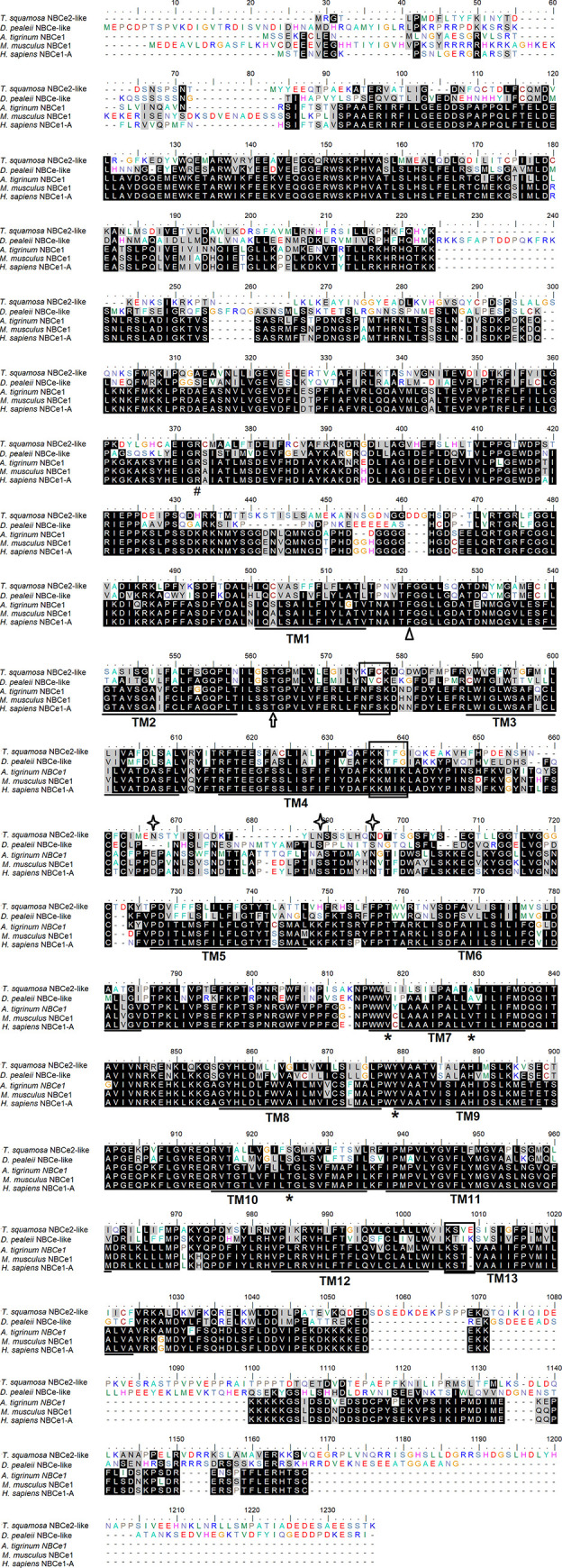
An alignment of the complete coding sequence of a homolog of electrogenic Na^+^-HCO_3_^−^ cotransporter 2 (NBCe2-like protein) obtained from the outer mantle of *Tridacna squamosa* with NBCe-like (ABF06444) sequence of *Doryteuthis pealeii*, NBCe1 (O13134.1) sequence of *Ambystoma tigrinum*, NBCe1 (O88343.2) of *Mus musculus*, and NBCe1-A (NP_001091954.1) of *Homo sapiens*. Similar/identical amino acid residues are shaded. The 13 predicted transmembrane regions (TM1-TM13) are underlined. Asterisks indicate the conserved residues important in electrical properties. Hash, arrow and open triangle denote the conserved residues essential for a functional NBCe. Boxes depict the putative DIDS-binding motifs. Diamonds denote residues predicted for N-glycosylation. The transmembrane regions were predicted using TOPCONS.

**Fig 2:**
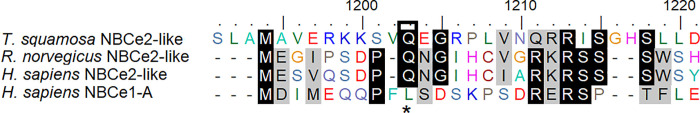
An aligment of the partial sequence of a homolog of electrogenic Na^+^-HCO_3_^−^ cotransporter 2 (NBCe2-like protein) obtained from the outer mantle of *Tridacna squamosa* with the partial sequences of NBCe2 of *Rattus norvegicus* (NP_997677.1) as well as NBCe2 (AAK26741.1) and NBCe1-A (NP_003750.1) of *Homo sapiens* in order to examine the basolateral targeting motif. Similar/identical amino acid residues are shaded. The asterisk indicates the coordinating residue for the basolateral targeting motif of NBCe1-A from human. The absence of the basolateral targeting residue in NBCe2-like of *T*. *squamosa*, as well as NBCe2 of *R*. *norvegicus* and *H*. *sapiens*, is highlighted by a box.

**Fig 3:**
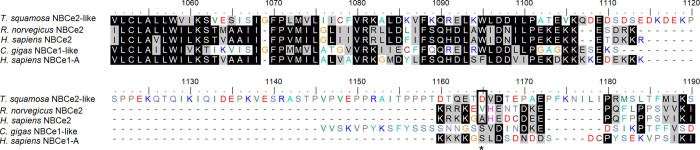
An aligment of the partial sequence of a homolog of electrogenic Na^+^-HCO_3_^−^ cotransporter 2 (NBCe2-like protein) obtained from the outer mantle of *Tridacna squamosa* with the partial sequences of NBCe2 of *Rattus norvegicus* (NP_997677.1) as well as NBCe2 (AAK26741.1) and NBCe1-A (NP_003750.1) of *Homo sapiens*, in order to examine the serine residue, which can undergo phosphorylation to change the ratio of Na^+^: HCO_3_^−^ transport. Similar/identical amino acid residues are shaded. The asterisk denotes the serine residue (position 1165 based on the multiple amino acid alignment) that can be phosphorylated to induce a shift in the Na^+^: HCO_3_^−^ stoichiometry from 1: 3 (efflux of HCO_3_^−^) to 1: 2 (influx of HCO_3_^−^). This serine residue (highlighted by a box) is present only in NBCe1-A of human (Ser^982^ based on the human sequence), but is lacking in NBCe2-like protein of *T*. *squamosa*, as well as NBCe2 of *R*. *norvegicus* and *H*. *sapiens*.

The *NBCe2-like* nucleotide sequence obtained from the outer mantle of *T*. *squamosa* was deposited into Genbank (MW821489). It comprised 3,399 bp, encoding a protein of 1,132 amino acids with a predicted molecular mass of 127.3 kDa (Fig 1). As NBCe2 is not well characterized, NBCe-like from *D*. *pealeii*, NBCe1 from *Ambystoma tigrinum*, NBCe1 from *Mus musculus*, and NBCe1-A from *Homo sapiens* were used to determine the essential residues and motifs in NBCe2-like protein of *T*. *squamosa*. NBCe2-like protein of *T*. *squamosa* contained 13 TMs, as well as various conserved characteristics of NBCe1 (Fig 1). These include (1) the DIDS-binding domain, (2) the residues essential for electrical properties (Gly^445^, Phe^460^, Leu^715^, Ala^726^, Tyr^776^, Ser^822^, Ala^850^ of *T*. *squamosa* NBCe2-like), (3) the arginine residue (Arg^285^ in *T*. *squamosa* NBCe2-like) located at the N-terminal region and involved in forming the HCO_3_^−^ tunnel, and (4) two threonine residues (Thr^431^ and Thr^474^ in *T*. *squamosa* NBCe2-like) required for a functional NBCe transporter. In addition, NBCe2-like protein of *T*. *squamosa* comprised three asparagine residues (Asn^575^, Asn^588^, and Asn^595^) that could undergo N-glycosylation (Fig 1). A comparison was made among the NBCe2-like protein obtained from the outer mantle of *T*. *squamosa* (this study), NBCe2 of *Rattus norvegicus* (NP_997677.1), as well as NBCe2 (AAK26741.1) and NBCe1-A of *H*. *sapiens* (NP_003750.1). Results indicate that NBCe2-like protein of *T*. *squamosa*, NBCe2 of rat and NBCe2 of human lacked the important leucine residue of the basolateral targeting motif (FL motif) of human NBCe1-A (Fig 2). Furthermore, these three sequences lacked a serine residue equivalent to Ser^982^ of human NBCe1-A (Fig 3).

### Immuno-localization of NBCe2-like protein in the outer mantle

**Fig 4:**
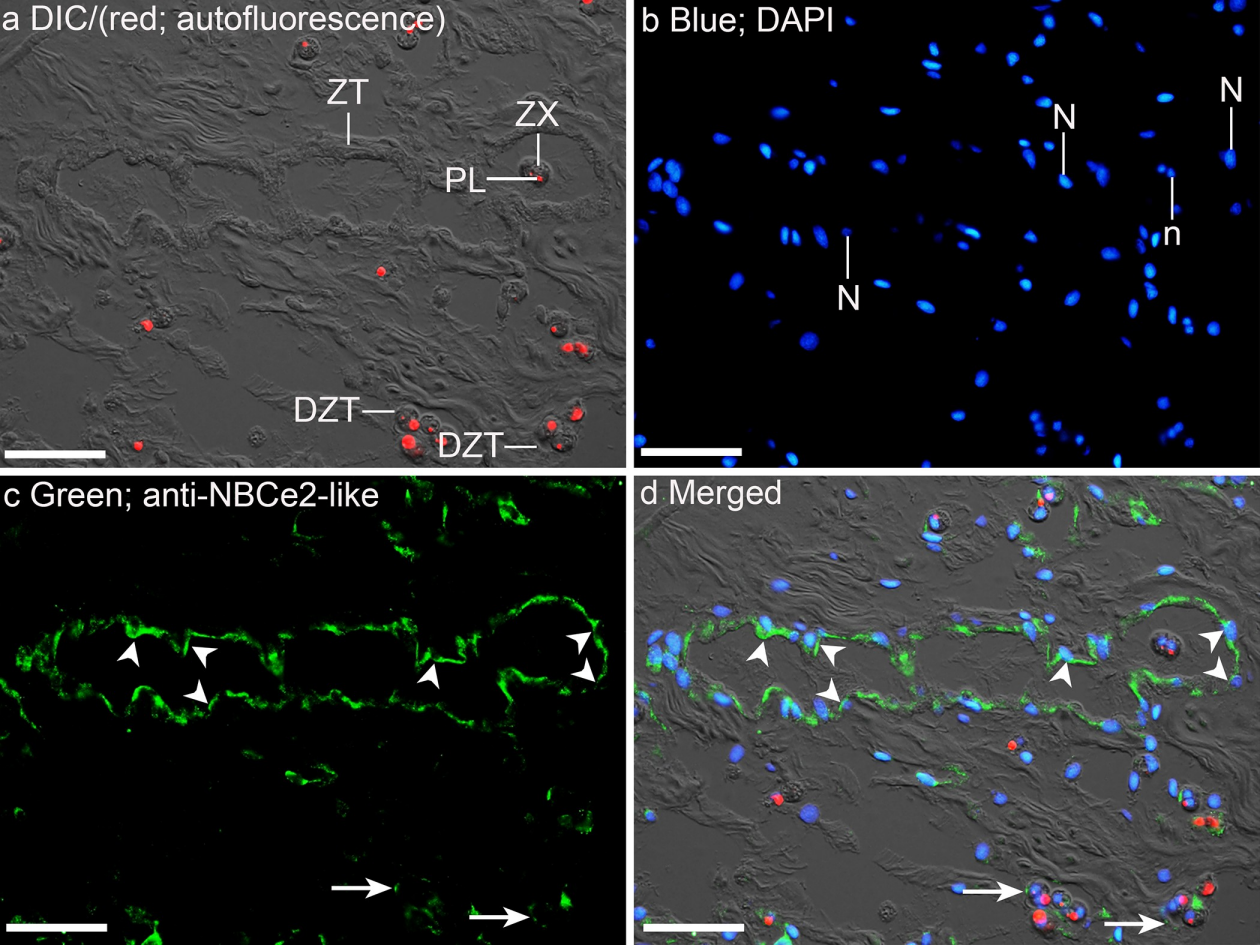
Immunofluorescence localization of a homolog of electrogenic Na^+^-HCO_3_^−^ cotransporter 2 (NBCe2-like protein) in the outer mantle of *Tridacna squamosa* exposed to light for 12 h (a to d). (a) A differential interference contrast image (DIC) showing the structures of zooxanthellal tubules (ZTs) and disrupted zooxanthellal tubules (DZTs) overlaid with the red channel showing autofluorescence of the plastids (PLs) of the symbiont dinoflagellates (zooxanthellae, ZX) in red. (b) Nuclei stained with DAPI in blue, whereby n represents the round nucleus of symbionts and N represents the elongated nucleus of ZTs, (c) NBCe2-like-immunofluorescence is displayed in green. (d) The DIC image is merged with the red, blue and green channels. Arrowheads in (c, d) indicate the apical staining (with reference to the position of the elongated nucleus) of NBCe2-like in the membrane of the epithelial cells of ZTs surrouding the ZX in the outer mantle. Arrowheads with tail in (c, d) indicate the faint NBCe2-like-immunolabeling of DZTs. HL, hemolymph. Scale bar: 20 μm. Reproducible results were obtained from three clams exposed to light.

It was essential to capture the nuclei of the tubular epithelial cells during immunofluorescence microscopy in order to identify the location of NBCe2-like protein in the tubular epithelium. The nuclei of these epithelial cells were elongated, which is unlike the round nuclei within the symbiotic dinoflagellates, and immunofluorescence detected between the elongated nucleus and the lumen of the zooxanthellal tubule would indicate an apical localization. Indeed, NBCe2-like-immunofluorescence was detected mainly along the apical membrane of epithelial cells of zooxanthellal tubules in the outer mantle of *T*. *squamosa* (Fig 4). Some epithelial cells of zooxanthellal tubules were disrupted during sectioning and sample preparation, resulting in the faint NBCe2-like-immunofluorescence (Fig 4). Results were reproducible with three biological replicates (n = 3). The validity of NBCe2-like immunolabelling was confirmed by the PCA (S1 Fig).

### Effects of light on the *NBCe2-like* transcript level and NBCe2-like protein abundance in the outer mantle

**Fig 5:**
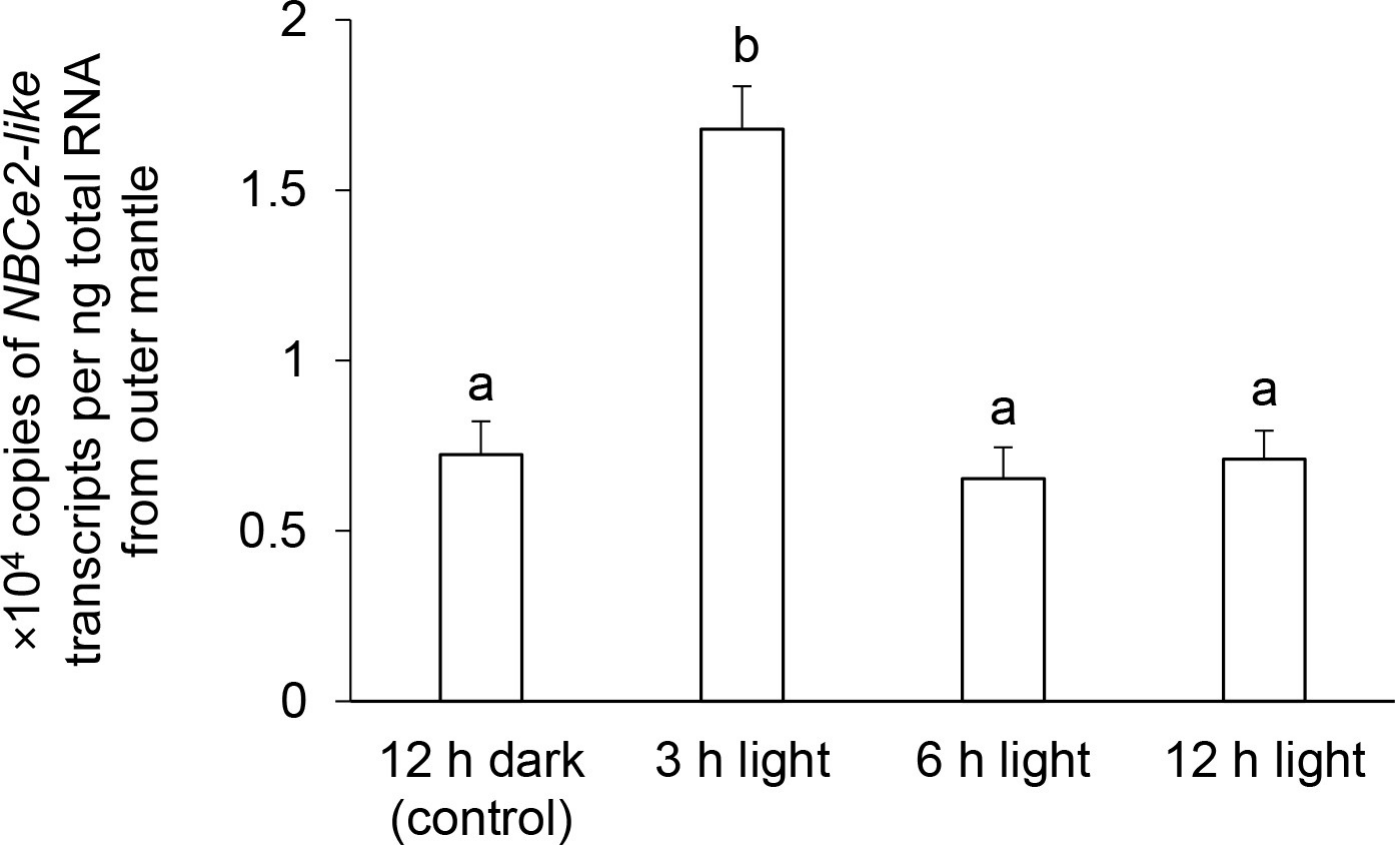
The transcript level (×10^4^ copies of transcript per ng of total RNA) of a homolog of *electrogenic Na*^*+*^*-HCO_3_^−^ cotransporter 2* (*NBCe2-like* gene) from the outer mantle of *Tridacna squamosa* kept in darkness for 12 h dark (control), or exposed to light for 3, 6, or 12 h. Results represent means + SEM (n = 5). Means not sharing the same letter are significantly different (p < 0.05).

**Fig 6:**
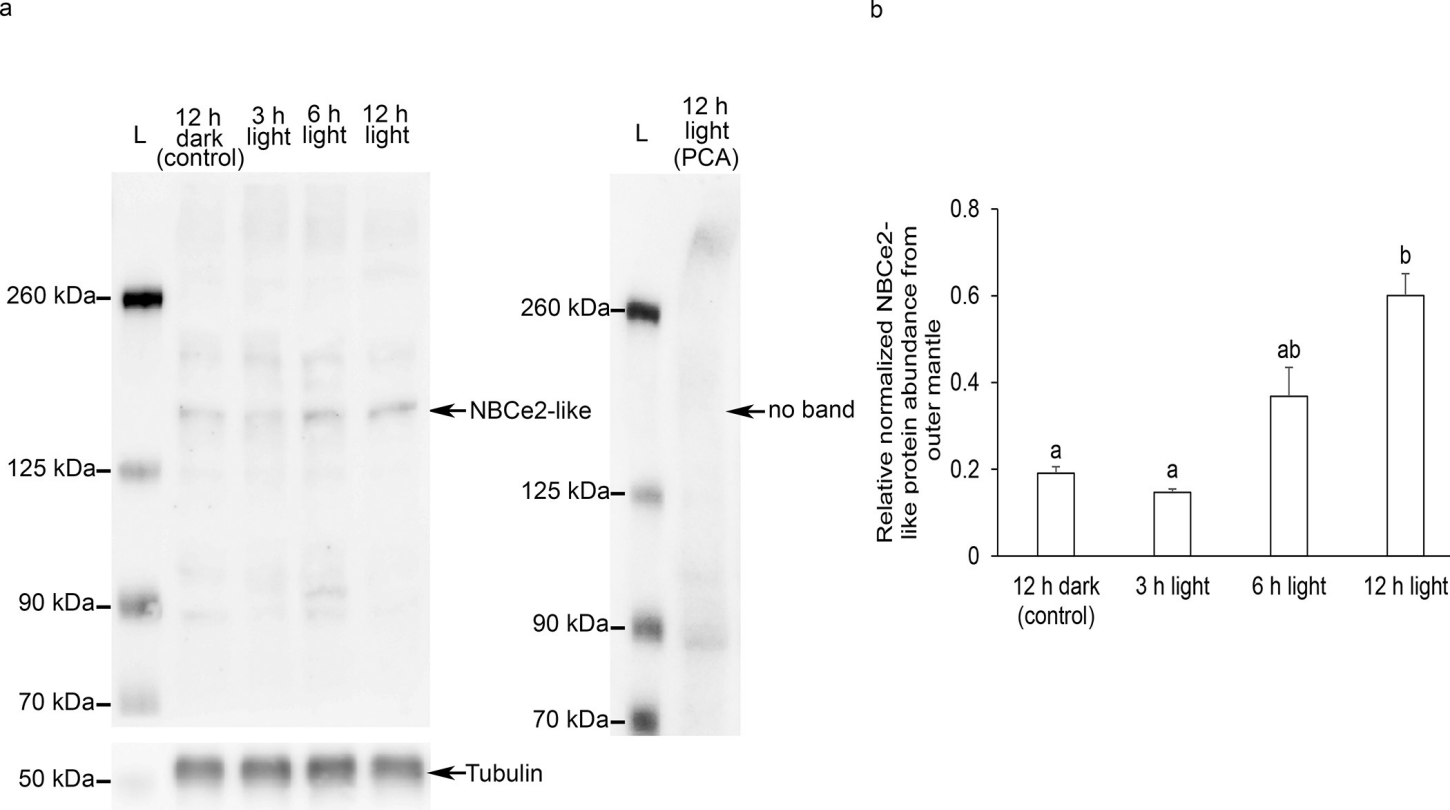
The protein abundance of a homolog of electrogenic Na^+^-HCO_3_^−^ cotransporter 2 (NBCe2-like protein) from the outer mantle of *Tridacna squamosa* exposed to 12 h of darkness (control) or 3, 6, 12 h of light. (a) Examples of the immunoblots of NBCe2-like, with or without the anti-NBCe2-like antibody neutralized by the immunizing peptide in a peptide competition assay (PCA), and the immunoblot of tubulin as the reference protein. (b) The optical density of the NBCe-like band for 100 μg protein was normalized with respect to that of tubulin. Results represent means + SEM (n = 5). Means not sharing the same letter are significantly different. (p < 0.05).

There was a significant increase (~2.3 fold) in the level of *NBCe2-like* transcript in the outer mantle of individuals exposed to light for 3 h as compared with that of the control kept in darkness for 12 h. It subsequently returned to the control level at hour 6 and hour 12 of light exposure (Fig 5). Western blotting revealed a band-of-interest that was slightly higher than 125 kDa as displayed by the molecular ladder. However, the retardation factor (Rf) plot estimated the molecular mass of the band-of-interest to be approximately 150 kDa, which was slightly higher than the mass of 127.3 kDa deduced from the NBCe2-like amino acid sequence. The difference between the estimated and deduced molecular mass of NBCe2-like could possibly be due to N-glycosylation of the three asparagine residues (Asn^575^, Asn^588^, and Asn^595^). Importantly, the abundance of NBCe2-like protein increased significantly in the outer mantle of *T*. *squamosa* exposed to light for 12 h as compared with the control kept in darkness (Fig 6).

## Discussion

Symbiotic dinoflagellates are fundamentally responsible for phototrophy in giant clam-dinoflagellate associations [20,37]. Therefore, light-enhanced C_i_ fixation by symbionts residing in the colorful outer mantle is foundational to many light-enhanced processes in various organs of the host [19]. Indeed, the outer mantle of *T*. *squamosa* possesses a light-enhanced CCM to increase the delivery of C_i_ from the hemolymph into the luminal fluid of the zooxanthellal tubules to benefit the photosynthesizing symbionts during illumination. In this study, we report the expression of an apical NBCe2-like protein in the epithelial cells of the zooxanthellal tubules in the outer mantle of *T*. *squamosa*. In mammals, NBCe1 and NBCe2 are differentiated primarily by their subcellular localization instead of molecular characteristics; NBCe1 has a basolateral localization [38–40] while NBCe2 is localized at the apical membrane [30,41–45]. Based on immunofluorescence microscopy, we had confirmed that NBCe2-like protein was indeed localized at the apical membrane of the tubular epithelial cells in the outer mantle of *T*. *squamosa*. As the expression levels of NBCe2-like protein was upregulated in the outer mantle by illumination, it could be the putative transporter involved in the increased translocation of HCO_3_^−^ from the epithelial cells into the luminal fluid of the tubular system to support the photosynthesizing symbionts.

### Molecular properties of NBCe2-like protein obtained from the outer mantle of *T*. *squamosa*

As NBCe2 is not well characterized, the human NBCe1-A was used as a comparison to determine the essential residues and motifs in NBCe2-like protein of *T*. *squamosa*. In human NBCe1-A, Thr^442^ (corresponding to Thr^431^ in NBCe2-like of *T*. *squamosa*) is responsible for the formation of an external gate for the ions to be transported [46]. The N-terminal region of human NBCe1-A possesses a functionally important residue, Arg^298^ (corresponding to Arg^285^ in NBCe2-like of *T*. *squamosa*). This residue is positioned in a tightly folded aqueous inaccessible region and participates in the formation of a ‘HCO_3_^−^ tunnel’ that can be disrupted by the R298S mutation [47,48]. As the N-terminal region can interact with TMs [49], R298S mutants may have a low efficiency in delivering HCO_3_^−^ to the ‘HCO_3_^−^ tunnel’ in the TMs. Several residues that contribute to the electrogenicity of NBCes [50] are conserved in NBCe2-like protein of *T*. *squamosa* (Gly^445^, Phe^460^, Leu^715^, Ala^726^, Tyr^776^, Ser^822^, Ala^850^).

The putative DIDS-binding motifs of NBCs consist of two Lys residues separated by two other amino acids (e.g., KXXK) [51–53]. The ends of TM 3, 5, and 12 may spatially form a DIDS-binding pocket [54]. NBCe2-like of *T*. *squamosa* has KFCK at TM3, KKTFG at TM5, and KSVE at TM12. In comparison, human NBCe1-A has KKMIK at the end of TM5 with two ‘disrupted’ motifs (NFSK near TM 3, and KSTV near TM 12). In spite of the disrupted DIDS motifs, NBCe1-A is still sensitive to DIDS. Moreover, the mutation of any one of the three TM5 Lys residues has little effect on irreversible DIDS inhibition [55]. Thus, it is possible that DIDS could bind reversibly to NBCe2-like of *T*. *squamosa* at TM5 and/or TM12, and then quickly reacts with a Lys residue to produce a permanent blockade.

N-glycosylation of NBCe1 is very common among mammals including human. Human NBCe1-A consists of 1,035 amino acids and has a predicted molecular mass of 116 kDa [51]. As it contains three predicted glycosylation sites (Asn^592^, Asn^597^, and Asn^617^), its molecular mass increases to ~ 130–145 kDa after glycosylation [56]. Mutation of the three glycosylation sites leads to de-glycosylation of human NBCe1, but the de-glycosylated NBCe1 retains its basic functions, although de-glycosylation might have affected its folding efficiency and/or stability [56]. Although only Asn^617^ of NBCe1-A from human is conserved in NBCe2-like protein of *T*. *squamosa* (Asn^595^), the latter also contains two other predicted glycosylation sites (Asn^575^ and Asn^588^). This could explain why the molecular mass of NBCe2-like of *T*. *squamosa* estimated by western blotting (~ 150 kDa) was apparently higher than that deduced from the amino acid sequence (127.3 kDa).

### NBCe2-like protein has an apical localization in the tubular epithelial cells and can therefore transport HCO_3_^−^ between these cells and the tubule’s luminal fluid

Immunofluorescence microscopy revealed that NBCe2-like was localized at the apical membrane of the epithelial cells that formed the zooxanthellal tubules in the outer mantle of *T*. *squamosa*. According to the literature, all NBCe1 have a basolateral localization in various epithelia (proximal renal tubule of human [38]; human pancreatic ducts [39]; intestinal tract of toadfish [40]) based on immunofluorescence microscopy. For human NBCe1, the C-terminal region consists of a basolateral-targeting FL motif [57]. By contrast, NBCe2 is localized in the apical membranes of many types of epithelial cell. These include the cholangiocyte in the bile duct [41], the cells of renal proximal tubules [30], the intercalated cells in renal collecting duct [42] and the uroepithelial cells in the renal pelvis [43] of human, as well as the cells in the choroid plexus epithelium (CPE) in the brain of rodent [44,45]. The only exception are human hepatocytes, in which NBCe2 is localized in the sinusoidal (basolateral) membrane [58,59], but the reason for this remains unknown at present.

A sequence alignment of NBCe2-like protein of *T*. *squamosa* with NBCe2 of rat, as well as NBCe2 and NBCe1-A of human reveals that all three NBCe2-like and NBCe2 sequences lack the L residue of the basolateral targeting FL motif of human NBCe1-A. It has been demonstrated that the substitution of Lys of the FL motif with Ala in the originally basolateral NBCe1 results in the retargeting of the mutant to the apical membrane of Madin-Darby canine kidney cells [60]. Hence, the molecular property of NBCe2-like protein obtained from *T*. *squamosa* corroborates its apical localization in the tubular epithelial cells as revealed by immunofluorescence microscopy. With such a subcellular localization, NBCe2-like protein is positioned to transport HCO_3_^−^ between these cells and the luminal fluid of the tubule.

### NBCe2-like protein probably catalyzed the efflux of HCO_3_^−^ from the epithelial cells

NBCes (NBCe1 and NBCe2) can transport Na^+^ and HCO_3_^−^ into or out of a cell. The co-transport of Na^+^ and HCO_3_^−^ involves the electrochemical potential gradients of both ions, and the direction of co-transport is defined by the transport ratio of Na^+^: HCO_3_^−^, that is, 1: 2 or 1: 3. Under normal circumstances, the electrochemical gradient of Na^+^ existing across the plasma membrane would drive the passive movement of Na^+^ into the cell, because the transmembrane electrical potential is normally inside negative and the extracellular concentration of Na^+^ is higher than the intracellular Na^+^ concentration. At a Na^+^:HCO_3_^−^ stoichiometry of 1:2, the direction of co-transport is governed by the electrochemical gradient of Na^+^, which drives both ions into the cell. However, at a Na^+^:HCO_3_^−^ stoichiometry of 1:3, Na^+^ and HCO_3_^−^ would be co-transported out of the cell as the direction of transport is defined by the electrochemical gradient of HCO_3_^−^, which is acting outward. Notably, the Na^+^: HCO_3_^−^ ratio can be changed from 1: 3 to 1: 2 by the phosphorylation of a Ser residue near the C-terminus.

For instance, NBCe1-A is expressed predominantly in the basolateral membrane of S1 and S2 proximal tubule cells in human kidney, and it operates in an efflux mode with a Na^+^: HCO_3_^−^ stoichiometry of 1:3 [38]. It mediates basolateral HCO_3_^−^ efflux from these tubule cells to the blood, contributing to the reabsorption of ~80% of the filtered HCO_3_^−^ in the lumen of the tubule. By contrast, in human pancreatic duct, the basolateral NBCe1-B operates in an influx mode with a Na^+^: HCO_3_^−^ stoichiometry of 1: 2 to absorb HCO_3_^−^ from the blood into the cell [60,61]. It has been established that phosphorylation of Ser^982^ at the C-terminal region of human NBCe1-A by protein kinase A (PKA) alters its Na^+^: HCO_3_^−^ stoichiometry of operation from 1: 3 to 1: 2 when transfected into mouse proximal convoluted tubule cells [62]. After Ser^982^ is replaced with Ala in human NBCe1-A, PKA fails to phosphorylate NBCe1-A to induce the Na^+^:HCO_3_^−^ stoichiometry shift from 1:3 to 1:2 [62].

**Fig 7:**
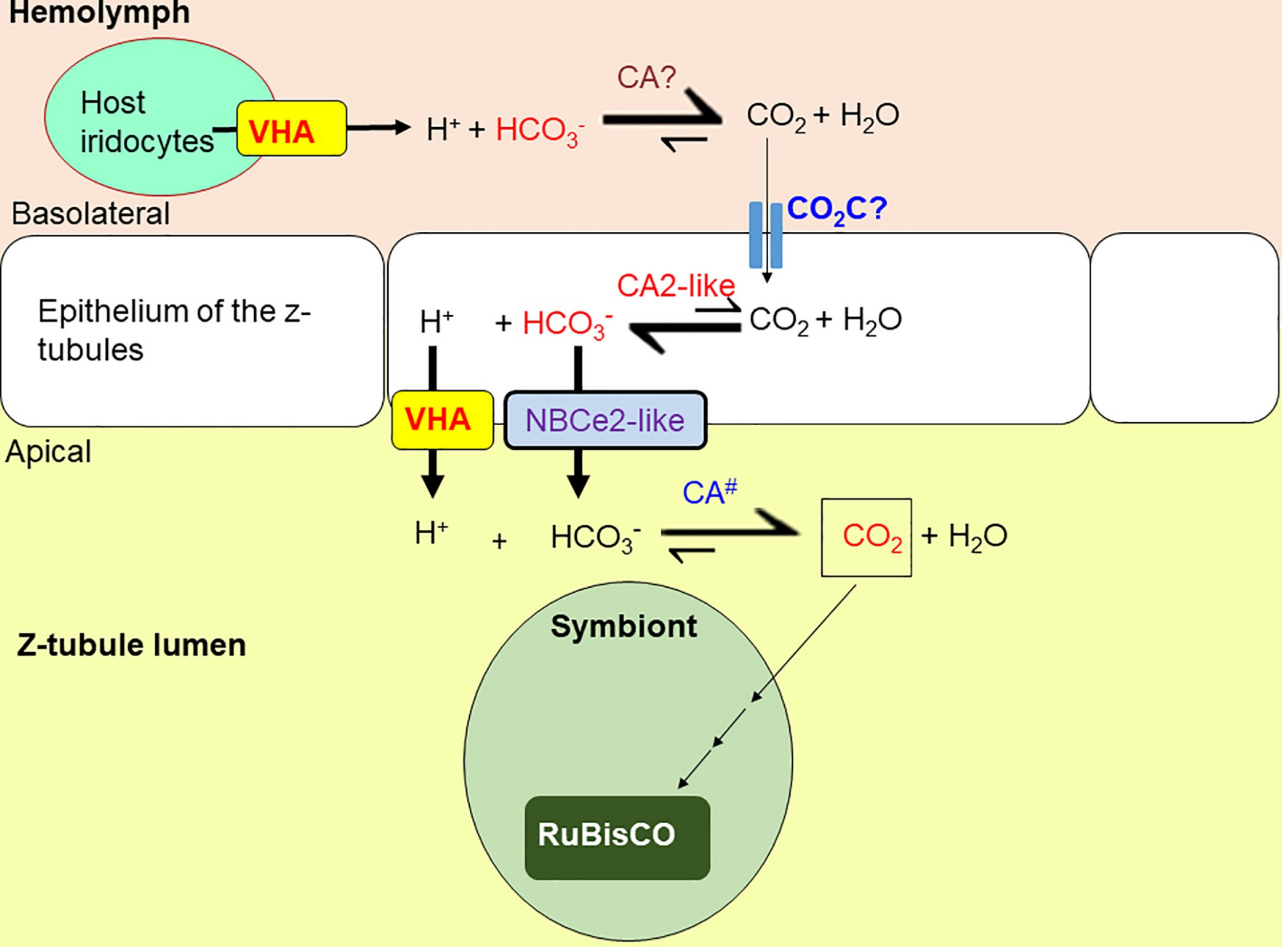
A proposed scheme for the role of a homolog of electrogenic Na^+^-HCO_3_^−^ cotransporter 2-like (NBCe2-like) in the host-mediated carbon-concentrating mechanism (CCM) to transport HCO_3_^−^ across the apical membrane of the epithelial cells of the zooxanthellal tubules (z-tubules) into the luminal fluid in the the outer mantle of *Tridacna squamosa*. The H^+^ secreted by the host irridocytes through vacuolar H^+^-ATPase (VHA) [24] could augment the dehydration of HCO_3_^−^ in the hemolymph to CO_2_ catalyzed by a hypothetical extracellular CA that is secreted by the host (CA?). CO_2_ could then permeate through an unknown CO_2_ channel (CO_2_C?) into the epithelial cells of zooxanthellal tubules, and be hydrated back into HCO_3_^-^ catalyzed by the cytosolic carbonic anhydrase 2-like (CA2-like) [25] in the cytoplasm. Subsequently, cytoplasmic HCO_3_^−^ could be transported through the apical NBCe2-like protein (this study) into the luminal fluid where the symbionts are residing. The epithelial cells that form the zooxanthellal tubules could secrete H^+^ through VHA localized at the apical membrane [24]. The secreted H^+^ could augment the dehydration of HCO_3_^−^ to CO_2_ in the luminal fluid catalyzed by a hypothetical extracellular carbonic anhydrase (CA^#^) of symbiont origin. Eventually, CO_2_ is absrobed by the symbionts and utilized by form II ribulose-1,5-bisphosphate carboxylase/oxygenase (RuBisCO) during photosynthesis [21].

Importantly, NBCe2-like protein of *T*. *squamosa*, as well as NBCe2s of *R*. *norvegicus* and human, lack Ser^982^ of human NBCe1-A (Fig 3). Therefore, it is logical to deduce that, the apical NBCe2-like protein of *T*. *squamosa* could function in a Na^+^: HCO_3_^−^ stoichiometry of 1: 3 to facilitate the efflux of HCO_3_^−^ from the cytoplasm of the tubular epithelial cells into the luminal fluid (Fig 7). This is in agreement with the consensus that NBCe2 operates in a Na^+^: HCO_3_^−^ stoichiometry of 1: 3 to promote the efflux of Na^+^ and HCO_3_^−^ in native tissues. Although *in vitro* patch-clamp studies on NBCe2-expressing embryonic kidney cells of humans (HEK-293) indicates that NBCe2 operates at a Na^+^: HCO_3_^−^ stoichiometry of 1: 2 [63], *in vivo* studies have suggested a Na^+^: HCO_3_^−^ stoichiometry of 1: 3 instead [31,33]. Furthermore, NBCe2 is posited to participate in HCO_3_^−^ efflux into cerebrospinal fluid (CSF) in choroid plexus of mouse brain, in order to regulate the pH of CSF during hypercapnia-induced acidosis [33]. Indeed, knockout of NBCe2 in the choroid plexus can lead to a net decrease and increase in base and acid extrusion, respectively, supporting the role of NBCe2 in HCO_3_^−^ efflux into CSF [31]. In mouse kidney, NBCe2 is expressed predominantly in renal connecting tubules (CNT) and cortical collecting tubules (CD) [63]. Knockout of NBCe2 in CNT and CD of mouse also decreases net base extrusion and increases net acid extrusion, suggesting that NBCe2 plays a role in mediating efflux of Na^+^ and HCO_3_^−^ in mouse renal tubules [64].

### Illumination increases the expression of NBCe2-like transcript and protein in the outer mantle in order to increase the supply of C_i_ to the photosynthesizing symbionts

Exposure to light for 12 h resulted in significant increases in the level of *NBCe2-like* transcript and abundance of /NBCe2-like protein in the outer mantle of *T*. *squamosa*. Notably, the upregulation of the transcript level was transient and occurred at hour 3, prior to the upregulation of NBCe2-like protein abundance. These results indicate that the expression of NBCe2-like protein *T*. *squamosa* is light-dependent and is regulated at both the transcriptional and the translational levels. Hence, it can be deduced that illumination could augment the capacity of HCO_3_^−^ extrusion into the luminal fluid through NBCe2-like in the outer mantle of *T*. *squamosa*. This aligns well with photosynthetic activity in the symbiotic dinoflagellates present in the outer mantle, which requires an increase in the supply of C_i_. It is noteworthy that illumination also leads to a significant increase in the protein abundance of form II Ribulose-1,5-bisphosphate carboxylase/oxygenase of symbiont origin in the outer mantle of *T*. *squamosa* [21].

### Summary

Giant clams are phototrophic because they harbor symbiotic dinoflagellates that can conduct photosynthesis during illumination. Hence, light-enhanced C_i_ fixation by symbionts is foundational to other light-enhanced processes in the host. This implies that the clam host must increase the uptake of C_i_ from the ambient seawater and the supply of C_i_ through the hemolymph to the photosynthesizing symbionts residing in the luminal fluid of the zooxanthellal tubules in the outer mantle. The host can augment the translocation of C_i_ from the hemolymph to the luminal fluid of the tubules through a light-dependent CCM in the outer mantle, which involves VHA [24] and CA2 [25] of the tubular epithelial cells and iridocytes (Fig 7). Irridocytes can secrete H^+^ to the hemolymph through VHA [24], to augment the dehydration of HCO_3_^−^ to CO_2_. CO_2_ can be transproted into the epithelial cells of zooxanthellal tubules and hydrated back to HCO_3_^-^ catalyzed by the cytosolic CA2-like [25]. The apical NBCe2-like protein of the epithelial cells (this study) can transport the cytoplasmic HCO_3_^−^ to the luminal fluid where the symbionts are residing (Fig 7). Notably, the expression level of NBCe2-like is enhanced by light so that the capacity of HCO_3_^−^ transport is upregulated to augment the supply of C_i_ to the photosynthesizing symbionts.

## Supporting information

S1 Fig Validation of a homolog of electrogenic Na^+^-HCO_3_^−^ cotransporter 2 (NBCe2-like) in the outer mantle of *Tridacna squamosa* exposed to 12 h of light by a peptide competition assay (PCA). (a) The differential interference contrast (DIC) image shows the morphology of the symbionts (zooxanthellae, ZX) and zooxanthellal tubules (ZTs) of outer mantle. Autofluorescence produced by the plastids (PLs) of the ZX in red. The nuclei are stained blue using 4’,6-diamino-2-phenlyindole (DAPI). n represents nuclei of ZX while N represents nuclei of ZTs in elongated shape. (b, c) The apical NBCe2-like staining is not present. Scale bar: 20 μm.
(DOCX)
