# Supplementary material for: The colorful mantle of the giant clam Tridacna squamosa expresses a homolog of electrogenic sodium: Bicarbonate cotransporter 2 that mediates the supply of inorganic carbon to photosynthesizing symbionts
Source: PLoS One. 2021 Oct 15;16(10):e0258519. doi: 10.1371/journal.pone.0258519 (PMC8519421; doi:10.1371/journal.pone.0258519)

**Supp. Fig. S1** Validation of a homolog of electrogenic Na^+^-HCO_3_^−^ cotransporter 2 (NBCe2-like) in the outer mantle of *Tridacna squamosa* exposed to 12 h of light by a peptide competition assay (PCA). (a) The differential interference contrast (DIC) image shows the morphology of the symbionts (zooxanthellae, ZX) and zooxanthellal tubules (ZTs) of outer mantle. Autofluorescence produced by the plastids (PLs) of the ZX in red. The nuclei are stained blue using 4’,6-diamino-2-phenlyindole (DAPI). n represents nuclei of ZX while N represents nuclei of ZTs in elongated shape. (b, c) The apical NBCe2-like staining is not present. Scale bar: 20 μm.


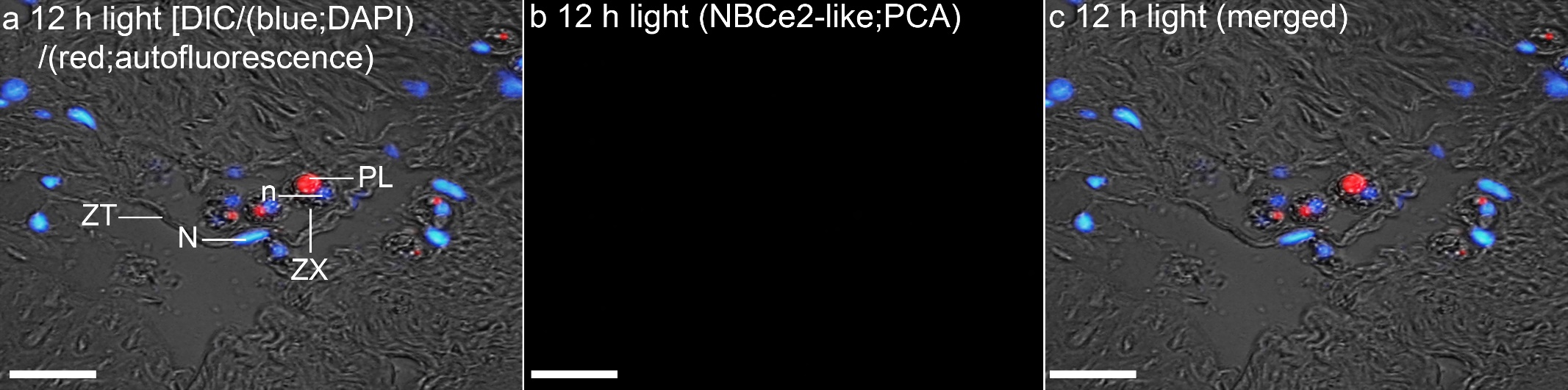

Supplement: S1 Fig — (a) The differential interference contrast (DIC) image shows the morphology of the symbionts (zooxanthellae, ZX) and zooxanthellal tubules (ZTs) of outer mantle. Autofluorescence produced by the plastids (PLs) of the ZX in red. The nuclei are stained blue using 4’,6-diamino-2-phenlyindole (DAPI). n represents nuclei of ZX while N represents nuclei of ZTs in elongated shape. (b, c) The apical NBCe2-like staining is not present. Scale bar: 20 μm. (DOCX) [file pone.0258519.s001.docx]
